# Optimization of Mesoporous Silica Nanoparticles through Statistical Design of Experiment and the Application for the Anticancer Drug

**DOI:** 10.3390/pharmaceutics13020184

**Published:** 2021-01-31

**Authors:** Min-Ki Kim, Do-Hyung Ki, Young-Guk Na, Hae-Soo Lee, Jong-Suep Baek, Jae-Young Lee, Hong-Ki Lee, Cheong-Weon Cho

**Affiliations:** 1 College of Pharmacy and Institute of Drug Research and Development, Chungnam National University, 99 Daehak-ro, Yuseong-gu, Daejeon 34134, Chungcheong, Korea; zkzkang@naver.com (M.-K.K.); youwewe@naver.com (D.-H.K.); youngguk@cnu.ac.kr (Y.-G.N.); hasoo0802@naver.com (H.-S.L.); jaeyoung@cnu.ac.kr (J.-Y.L.); 2 Department of Herbal Medicine Resource, Kangwon National University, Samcheok-si 25949, Gangwon-do, Korea; jsbaek@kangwon.ac.kr; 3 Department of Bio-Health Convergence, Kangwon National Univerisity, Chucheon 24341, Gangwon-do, Korea; 4 Animal Model Research Group, Jeonbuk Branch, Korea Institute of Toxicology (KIT), Jeongeup 53212, Jeollabuk-do, Korea

**Keywords:** mesoporous silica nanoparticles, doxorubicin, design of experiment, Box–Behnken design, optimization

## Abstract

The synthesis process or composition of mesoporous silica nanoparticles (MSNs) affects the physicochemical properties. Using these properties, MSNs were synthesized through the Box–Behnken design (BBD) among statistical experimental methods. The effect of the amounts of synthetic reagents, hexadecyl triethyl ammonium bromide (CTAB), tetraethyl orthosilicate (TEOS), and 2 N sodium hydroxide (NaOH), was studied using the reaction surface design. Surface area, particle size, and zeta potential were set as response values. The physicochemical properties of the optimized MSNs were evaluated, and the effect as a drug delivery system was evaluated by loading doxorubicin hydrochloride (DOX). Nano-sized MSNs were successfully prepared with 0.617 g of CTAB, 8.417 mL of TEOS, and 2.726 mL of 2 N NaOH and showed excellent physicochemical properties. The optimized MSNs showed negligible toxicity in MCF-7 cells. The drug release profile from DOX-loaded MSNs (MSN@DOX) showed an increased rate of release with decreasing pH of the medium, with the release profile sustained for 48 h. In the cytotoxicity test, the sustained drug release mechanism of MSN@DOX was confirmed. This study proposed a new statistical approach to the synthesis of MSNs.

## 1. Introduction

In the early 1990s, a new silica-based material called mesoporous silica was discovered. After that, mesoporous silica nanoparticles (MSNs) such as MCM-41, MCM-48, and SBA-15 with pore size of 2–10 nm, and 2D hexagonal and 3D cube structural properties have attracted great attention in various fields [1]. Recently, MSNs are actively being studied in the field of biomedicine, especially drug delivery systems [2,3]. Among the various nanomaterials, MSNs have become the next-generation inorganic material platform for biomedical applications [4,5,6]. In particular, MSNs are being actively studied in the field of drug delivery systems due to their unique mesostructural properties such as large surface area, large pore volume, adjustable particle size, easy surface functionalization, high stability, and good biocompatibility [7]. In addition, the physical structure and surface of MSNs can be easily modified to control pore size, surface area, and particle size, and can be chemically adjusted to optimize drug loading and release. The main advantage is that physical properties of MSNs can be controlled and changed depending on the synthetic process and materials [8,9]. To use MSNs as an ideal drug carrier, their stability, small particle size, and uniformity must be ensured [10]. In addition, the surface area and pore volume must be maximized to load large amounts of drug [11]. These parameters can be controlled by varying the composition of various components such as pH adjusters, surfactant concentrations, silica sources, and organic solvents and polymers [12]. Many studies have consistently presented ways to control the physical form of MSNs [13,14]. However, rather than using a variety of additional polymers and excipients, the method of controlling their physical form by varying their proportions in the basic synthetic reagents of MSNs will be the more efficient and preferred method. Various silica-based reagents, such as sodium silicate, tetramethylammonium silicate, and tetraethyl orthosilicate (TEOS) have been used as silicon sources for MSNs. Quaternary ammonium surfactants such as CTAB and cetrimonium chloride have mainly used as templates [8]. Because the properties of MSNs are influenced by many factors, it is important to control them to ascertain if the synthetic factors of MSNs affect their physical properties [7]. A statistical design approach, such as in the experimental design, was applied to understand the interaction between factors affecting the physical properties of MSNs according to changes in the MSN synthetic reagents. In particular, the Box–Behnken design (BBD) was used. The BBD is less costly but more efficient than a central composite design consisting of the same number of factors because it has fewer design points [15,16].

DOX is an anthracycline antibiotic commonly used in breast cancer chemotherapy [17]. DOX is a common anticancer agent which inserts into the DNA strand to interfere with tumor cell growth. Despite the advantages associated with DOX, there are obvious problems with side effects [18]. Anaphylaxis, heart failure, tissue necrosis at the injection site, and leukemia are typical examples. In serious cases, heart failure occurs after DOX administration, and the mortality rate for one year can reach 50%. In addition, in the absence of specific targeting, patients treated with DOX suffer [19]. DOX is a biopharmaceutics classification system (BCS) class 3 drug that is highly water-soluble and can be easily cross-linked with hydrophilic MSNs without complex bonding [4]. To reduce the side effects of DOX and to increase bioavailability, the MSNs would be beneficial as drug carriers for the delivery of DOX.

This study was performed to develop MSNs with a large surface area, small particle size, and low zeta potential value. The range of factors used for MSN optimization was selected through preliminary experiments. MSNs were optimized using BBD, a statistical experimental design method. The physicochemical properties of the optimized MSNs were investigated. Interestingly, there is no study about optimized MSN as a drug carrier of DOX through design of experiment. Thus, this study aims to optimize MSNs as a drug carrier using a statistical approach.

## 2. Materials and Methods

### 2.1. Materials

TEOS, CTAB, dimethyl sulfoxide (DMSO), 3-(4,5-dimethylthoazol-2yl)-2,5-diphenyl-2H-tetrazolium bromide (MTT), carbamazepine, and high-performance liquid chromatography (HPLC) grade acetonitrile (ACN) were purchased from Sigma Aldrich (St. Louis, MO, USA). Ethanol, toluene, NaOH, hydrochloric acid (HCl, 36–38 *v*/*v*%), and formic acid (>99.0%) were purchased from Samchun Pure Chemical (Pyungtaek, Korea). Human breast cancer cell (MCF-7) line was obtained from Korean cell line bank (Seoul, Korea). Dulbecco’s modified Eagle’s medium (DMEM), fetal bovine serum (FBS), penicillin–streptomycin, and trypsin–EDTA were purchased from Gibco^®^ BRL (Gaithersburg, MD, USA). DOX was provided as a gift from Korea United Pharm Inc. (Seoul, Korea).

### 2.2. Synthesis of MSNs

MSNs were synthesized following the modified Stöber method [20,21], though some parameters were changed according to the Box-Behnken design. Typically, 0.5 mg of CTAB was dissolved in 480 mL of distilled water, and then NaOH (3.5 mL, 2 N) was added to the aqueous solution of CTAB. The mixture was heated at 80 °C and stirred vigorously at 600 rpm. After the reaction mixture was stabilized at 80 °C for 30 min, 5 mL of TEOS was added dropwise at 0.33 mL/min. Subsequently, vigorous stirring for 2 h produced the MSNs suspension. The precipitate was obtained using centrifuge at 15,000 rpm for 20 min and washed out using ethanol and distilled water to eliminate unreacted materials. To completely remove the surfactant, the obtained residue was dispersed in 100 mL ethanol/HCl (1:8, *v*/*v*) for 20 min under ultrasound and refluxed at 60 °C for 6 h. It was then centrifuged at 15,000 rpm for 20 min and washed 3 times with ethanol and distilled water. The obtained residue was dried in an oven at 40 °C for more than 12 h to obtain the MSN powder.

### 2.3. Determination of Synthetic Reagent Amount

A simple preliminary experiment was conducted to determine the amount of CTAB, TEOS, and 2 N NaOH to use in the synthesis of the optimized MSNs. The synthesis method was carried out as described in the previous section “Synthesis of MSNs”. Then, 0.5 g of CTAB, 5 mL of TEOS, and 3.5 mL of 2 N NaOH were set as the basic amounts and MSNs were synthesized by changing the amounts of CTAB (0.125, 0.25, 0.5, 1.0, or 1.5 g), TEOS (1.25, 2.5, 5, 10, or 15 mL), and 2 N NaOH (0.88, 1.75, 3.5, 7.0, or 10.5 mL). The particle size of the synthesized MSNs was confirmed as described in Section 2.2. The structural properties of MSNs were analyzed by small angle X-ray diffraction (SAXRD) as described in the SAXRD section.

### 2.4. Optimization of MSNs

BBD is suitable for optimizing parameter levels through analysis of response surface design [22]. The experimental design and statistical analysis of BBD were conducted using the Design Expert^®^ 11 software (Sta-Ease Inc., Minneapolis, MN, USA). Experiments with BBD were designed to have 3 factors and 3 responses (Table 1). Factor selection and ranges were based on screening with CTAB amount (X_1_, g), TEOS volume (X_2_, mL), and 2 N NaOH volume (X_3_, mL) set in the ranges shown in Table 1. Responses according to the factors were set to the target response values for the maximum surface area (Y_1_), minimum particle size (Y_2_), and minimum zeta potential (Y_3_). Through BBD, 17 experiments with different factor values were designed, and the optimized response was fitted to one of the linear, 2-fi, quadratic, or tertiary models. The model presented for each response was analyzed by ANOVA. It was verified through variables consisting of the lack of p-value, the squared correlation coefficient (R^2^), and precision. As a result, MSNs with optimized response values were synthesized through a statistical model.

#### 2.4.1. Surface Area

The surface area of MSNs was measured using an ASAP 2420 analyzer (Micromeritics, Norcross, GA, USA). In brief, the sample was stabilized at 200 °C for 6 h, then the surface area was measured at −196.15 °C and calculated using the Brunauer–Emmett–Teller (BET) method [23].

#### 2.4.2. Particle Size and Zeta Potential

The particle size and zeta potential of MSNs was measured using ELS-Z2 (Osaka, Japan). About 5 mg of MSNs was suspended in 10 mL of PBS buffer (10 mM, pH 7.4) and sonicated for 5 min. The prepared suspension was measured at a 90° angle at 25 °C. In addition, before the measurement, all samples were properly diluted with distilled water. The sample was placed into a cuvette and then monitored with the ELS analyzer. The parameters were measured 50 times for each sample.

### 2.5. Physicochemical Properties of MSNs

Based on the BBD, the optimal composition of MSNs was selected and optimized MSNs were synthesized using 0.617 g of CTAB, 8.417 mL of TEOS, and 2.726 mL of 2 N NaOH. The physical properties of the MSNs were investigated.

#### 2.5.1. Morphology

The morphology of the MSNs was observed by field emission scanning electron microscopy (FE-SEM; FEI, Magellan 400, Hillsboro, OR, USA). The MSN samples were placed on carbon tape and made electrically conductive by coating with a thin layer of OsO4 in a vacuum. Then, the morphology of MSNs was observed by FE-SEM and the specific mesopore structure of MSNs was observed by FE-TEM (Tecnai G2 F30 S-Twin, FEI, Hillsboro, OR, USA). MSNs were properly diluted, dispersed with ethanol, and placed on carbon-coated 400 mesh copper grids. The grids were dried at room temperature (RT), and MSNs were imaged with FE-TEM operating at the accelerated voltage of 300 kV.

#### 2.5.2. SAXRD and Fourier Transform Infrared Spectroscopy

SAXRD analysis was performed to identify specific hexagonal two-dimensional peaks that appear only in MSN structures. SAXRD patterns were recorded in NANOPIX (RIGAKU, Tokyo, Japan) at an angle of 2θ and a scanning speed of 0.005°/s in the range of 0–6.5°.

Fourier transform infrared (FT-IR) spectroscopy was performed to confirm the binding that occurs during MSN synthesis. The FT-IR peak was confirmed using an ALPHA-P FT-IR spectrometer (Bruker Optics Inc., Billerica, MA, USA) in the range of 600 to 4000 cm^−1^ at RT. 

#### 2.5.3. Nitrogen Adsorption–Desorption Isotherm

To measure the surface area and pore size of MSNs, which are porous structures, the adsorption–desorption isotherm of nitrogen was measured according to the method described in the Section 2.4.2. The surface area was calculated by the BET method, and the pore size and distribution curves were obtained using the Barrett–Joyner–Halenda (BJH) method [24].

#### 2.5.4. In Vitro Degradation

In vitro degradation tests were performed to predict the in vivo degradation patterns of MSNs. The test was performed by dispersing 5 mg of MSNs in 30 mL of simulated body fluid (SBF, pH 7.4) and 30 mL of rat plasma, individually. The samples were stirred at 37 °C and 50 rpm. Each sample was collected at 0, 24, 48, and 72 h, and observed by TEM. The SBF was prepared using a previously reported method [25]. Briefly, 7.996 g NaCl, 0.350 g NaHCO_3_, 0.224 g KCl, 0.228 g K_2_HPO_4_·3H2O, 0.305 g MgCl_2_·6H_2_O, 0.278 g CaCl_2_, 0.071 g Na_2_SO4, 6.057 g tris(hydroxymethyl)aminomethane, and 20 mL of 2 M HCl were dissolved in 750 mL of distilled water. Then, the pH of the solution was adjusted to 7.4 at 37 °C with 1 N HCl, and the SBF was diluted to 1 L with distilled water.

### 2.6. Characterization of MSN@DOX

DOX was selected as a drug model for applying MSNs to drug delivery systems. Here, DOX-loaded MSNs (MSN@DOX) were developed and evaluated by assessing the drug loading, in vitro drug release, cytotoxicity, and cellar uptake of MSN@DOX.

#### 2.6.1. HPLC Method

HPLC analysis of DOX was performed with the Agilent 1100 HPLC system (Agilent Technology, Santa Clara, USA) equipped with a UV detector. The column used was XterraTM RP C18, 5 μm × 4.6 mm × 250 mm, and the column temperature was maintained at 40 °C. The mobile phase was prepared with ACN and 10 mM NaH_2_PO_4_ (pH 4.0, phosphoric acid) at a ratio of 70:30. The flow rate was set to 1 mL/min and the injected volume was 20 μL. The detection wavelength was set to 480 nm, respectively. The encapsulation efficiency (*EE*) and loading capacity (*LC*) of DOX were calculated through the following equation.

$$\begin{array}{c} EE\;\left(\%\right)=\;\left[\left(\frac{Amount\;of\;DOX\;loaded\;into\;MSNs}{Initial\;amount\;of\;DOX}\right)\right]\;\times \;100\\ LC\;\left(\%\right)=\;\left[\left(\frac{Initial\;amount\;of\;DOX-\;amount\;of\;DOX\;supernatant}{Amount\;of\;MSNs+amount\;of\;DOX\;}\right)\right]\;\times \;100 \end{array}$$

#### 2.6.2. Drug Loading

DOX was loaded into the MSNs using a diffusion–filling–precipitation method [26]. Briefly, various amounts of DOX and 10 mg of MSNs were mixed in 5 mL of distilled water, and stirred slowly for 24 h under protection from light at RT. The mixture was adjusted to pH 7.8 by adding a dibasic sodium phosphate solution (0.1 mol/L), and a desalination process was induced. The DOX molecules adsorbed through the desalination process were precipitated and adsorbed into MSNs.

Each sample was centrifuged at 12,000 rpm for 10 min to collect MSN@DOX, and then washed 3 times with distilled water to remove unreacted material. Then, MSN@DOX powder was obtained through a lyophilization process. The amount of DOX in MSN@DOX was determined by measuring the supernatant obtained through centrifugation in the washing process by the HPLC method.

#### 2.6.3. In Vitro Drug Release

To confirm the release profile of DOX from MSN@DOX, an in vitro drug release test was conducted in PBS solutions with pH values of 5.0, 6.8, and 7.4. Briefly, a 1 mg/mL MSN@DOX suspension containing 2 mg of DOX was added to a 6–8 kDa dialysis bag, and stirred in a tube including 40 mL of PBS at 37 °C at 100 rpm. The drug release test was conducted for 48 h, and at predetermined time points, 0.5 mL of the sample was collected by filtration through a 0.45 μm filter. The same volume of fresh PBS was supplied to maintain the sink condition. The DOX concentration of the sample was determined by the HPLC method as in the Section 2.6.1.

### 2.7. Cell Study

#### 2.7.1. Cell Culture

Human breast cancer (MCF-7) cells were maintained in DMEM supplemented with 10% (*v*/*v*) FBS and 1% (*v*/*v*) penicillin–streptomycin under 5% CO_2_ at 37 °C.

#### 2.7.2. Cytotoxicity

MSN cytotoxicity was confirmed in MCF-7 cells using MTT assay. MCF-7 cells were seeded in 96-well plates in a cell culture medium at a density of 2.0 × 10^4^ cells/well (100 μL) and incubated for 24 h. Then, the DMEM was removed and washed carefully with pH 7.4 PBS. MSNs, MSN@DOX, and free DOX were dispersed in 1% PBS with 1% (*v*/*v*) DMSO and diluted with medium to add DOX at various concentrations ranging from 0.1 to 50 μg/mL (100 μL) to each well. After incubation for 24, 48, and 72 h, 30 μL of MTT solution (5 mg/mL) was added to each well and the cells were incubated at 37 °C for 3 h. After 3 h, the medium was removed, and MTT-formazan crystals were dissolved in 200 μL of DMSO. The absorbance of each well was measured at a wavelength of 565 nm using a microplate reader (Infinite M200 PRO; Tecan Trading AG, Männedorf, Switzerland). The cell viability of MCF-7 cells for different concentrations of MSNs was calculated using the following equation.

$$Cell\;viability\;\left(\%\right)=\frac{OD_{sample}}{OD_{control}}\times 100$$

#### 2.7.3. Cellular Uptake

MCF-7 cells were seeded in 12 well plates at a density of 1 × 10^4^ per plate and incubated in a DMEM medium for 24 h at 37 °C. Subsequently, the culture medium was replaced with PBS solutions containing MSNs, MSN@DOX, and free DOX. After 3 h of incubation, cells were washed 3 times with PBS solution, and cellular uptake of MSN@DOX was evaluated using a fluorescence microscope (EVOS M500; Invitrogen, CA, USA).

## 3. Results and Discussion

### 3.1. Synthesis of Optimized MSN

#### 3.1.1. Selection of Synthetic Reagent Range

**Figure 1:**
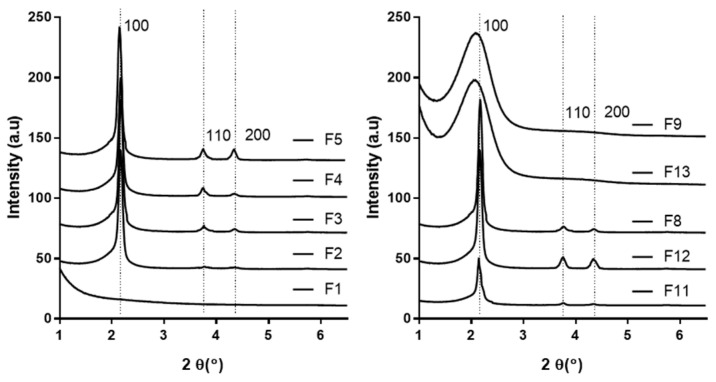
SAXRD patterns of the various mesoporous silica nanoparticles (MSNs). The peaks of 100, 110, and 200 hkl shown in the table represent specific peaks in the hexagonal structure of MSNs.

Before the optimization of MSN, the amounts of CTAB, TEOS, and 2 N NaOH were determined. The composition, particle size, and structure ordering of each sample are shown in Table 2. The structure ordering was determined through the presence or absence of particle formation and the SAXRD pattern (Figure 1).

In general, CTAB was used as the template for micelle formation during MSN synthesis [27]. White powder was formed in MSNs (F1, F2, F3, F4, and F5) using various amounts of CTAB. However, as shown in Figure 1, F1 did not show peaks corresponding to the 100, 110, and 200 hkl Miller indices. This is a characteristic peak of MCM-41 (mobile configuration of substance number 41) MSNs, showing a regular hexagonal pore structure [28]. When a small amount of CTAB was used (F1), it did not sufficiently form the mesoporous structure of MSNs. On the other hand, the mesostructure of F5 was non-uniform due to excessive CTAB [29].

TEOS acts as a silica precursor for the synthesis of MSNs, and its concentration affects the rate of seed growth and nucleation [30]. When a small amount of TEOS (F6, F7) was used, particles were not formed. Increasing the amount of TEOS (F3, F8) resulted in larger MSN sizes. In addition, although white powder was formed in F9 using 15.00 mL, it was confirmed that the specific structure of MSNs was not formed through SAXRD. It can be seen that there was insufficient TEOS in F6 and F7 to form the structure of MSNs; no particles were formed. On the other hand, in F9, which used a high silica precursor concentration, the hydrolysis of TEOS was incomplete. This resulted in a reagent residue in the product, meaning the mesostructure could not be confirmed, and the particle size could not be observed [13].

NaOH was used as a pH regulator and catalyst for the synthesis of MSNs [31]. Silica affects silane hydrolysis and siloxane condensation depending on pH [32]. Silica can exist stably at high pH due to the strong interaction between silicate–cationic surfactants. However, it remains unstable at low pH [33]. Here, we evaluated the particle size and structure order of MSNs by increasing the amount of NaOH (F10, F11, F3, F12, and F13). When a low amount of NaOH was used, particles were not formed. F13 using 10.5 mL produced a white powder, but it was confirmed that the MSN structure was not formed through SAXRD. This indicates that MSN particles were not generated due to the condensation reaction at a low pH and low concentration of NaOH at a slow hydrolysis rate of TEOS, but that the aggregated particles were formed due to excessive hydrolysis of TEOS at high pH [8]. As a result, the synthesis ranges were set to 0.25–1.0 g CTAB, 5.0–10.0 mL TEOS, and 1.75–7.0 mL 2N NaOH.

#### 3.1.2. Optimization of MSNs

Optimized MSNs were formulated using the BBD statistical analysis that was conducted to determine the relationship between the proposed model and the response through the Design Expert^®^ 12 software (Table 3). Surface area (Y_1_), particle size (Y_2_), and zeta potential (Y_3_) were crucial responses in the optimization of MSNs with excellent physical properties for application to drug delivery systems. The rationality of each reaction choice is as follows. 

A high surface area (Y_1_) means that MSNs have mesoporous structure, and the larger the surface area, the higher the drug load. The correlation between the surface area of MSNs and the amount of drug loading was investigated by prior studies. Particle size (Y_2_) and zeta potential (Y_3_) were chosen as response values with the purpose of their minimization. In general, smaller-sized MSNs are required to avoid immune responses by the retinal endothelial system of the liver and spleen. Zeta potential analysis is essential because it predicts the physical stability of the nanosuspension. Since MSNs carry a negative charge, the zeta potential of a large negative value in nanoparticles can predict high stability.

Table 4 shows the proposed models and parameters through interactions for response surface analysis. Those presented for each of surface area (Y_1_), particle size (Y_2_), and zeta potential (Y_3_) were quadratic, linear, and two-factor interaction models, respectively. To determine the fit of the statistical model, the statistical parameters of the lack of fit, p-value, and the squared correlation coefficient were checked [34]. In the proposed model, the sequential p-values are significant in the 95% confidence interval, meaning that the error is less than 5% [35]. The model’s lack of fit p-values exceeded 5%, making it suitable for explaining the model’s reliability and association [36]. The R^2^, adjusted R^2^ values, and the predicted R^2^ values indicate the degree to which the response of the proposed model matches the experimental data. In all reactions, R^2^ is higher than 0.9, and the difference between them is lower than 0.2, meaning that the experimental results are statistically significant [37,38]. 

**Figure 2:**
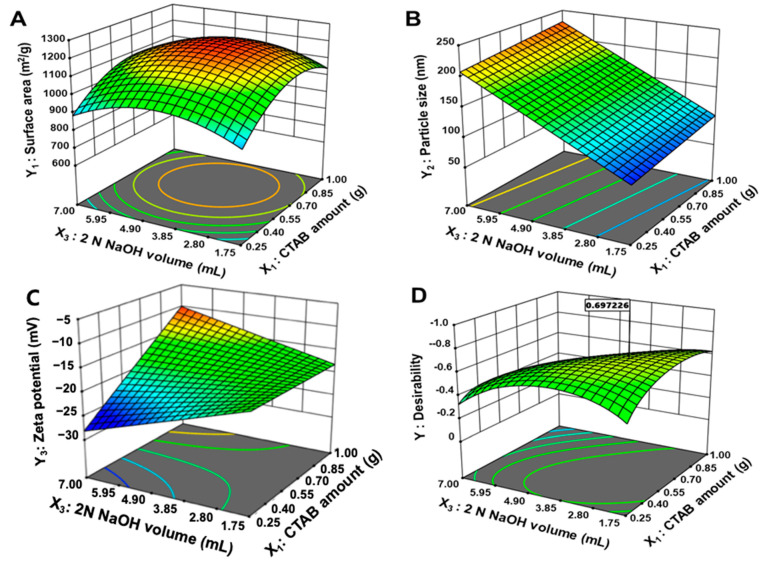
Three-dimensional surface plots of responses. (**A**), Y1: surface area; (**B**), Y2: particle size; (**C**), Y3: zeta potential; (**D**), plot of desirability value.

The interactions within the factors were described by three-dimensional diagrams along with coded equations. In three-dimensional plots (Figure 2), the X2 value was fixed with 7.5 mL because the low and high value of TEOS obstructed the formation of MSN structure and, also, it negatively affected all responses. Figure 2A–C and Table 5 show a three-dimensional plot of response surface analysis with response values within the set ranges. The confines of surface area (Y_1_), particle size (Y_2_), and zeta potential (Y_3_) were 818.5 to 1215.3 m^2^/g, 75.5 to 243.4 nm, and −25.7 to −9.3 mV, respectively.

Table 6 shows the coded spinning equation of the presented model. In the case of surface area (Y_1_), positive coefficients were found for CTAB amount (X_1_), TEOS amount (X_2_), and surface area (X_3_). This indicates that the surface area (Y_1_) was increased as each factor increases. In particular, the CTAB amount (X_1_) showed a superior effect on surface area (Y_1_) than other factors. According to the Vazquez et al., the addition of CTAB induces silica mesoporous structure growth following an Ostwald ripening mechanism [39]. In particular, CTAB plays an important role in the formation of agglomerates, changing the TEOS hydrolysis. In addition, it has been reported that the use of CTAB led the increase of surface area (Y_1_). As CTAB amount (X_1_), TEOS amount (X_2_), and NaOH volume (X_3_) increase, the particle size (Y_2_) increase with a synergistic effect. Among the factors, NaOH volume (X_3_) had the most influence on particle size (Y_2_). Increasing the pH of the aqueous phase with an increase in 2 N NaOH can lead to larger particle formation due to condensation of TEOS, which has a faster hydrolysis rate. Zeta potential (Y_3_) showed an increasing effect as CTAB amount (X_1_) increased; however, as TEOS amount (X_2_) and NaOH volume (X_3_) increased, zeta potential (Y_3_) decreased. Among them, CTAB amount (X_1_) had the most influence on the zeta potential (Y_3_), and a CTAB amount (X_1_) ranging from 0.5 to 1.5 g can lead to stable particle formation and distribution through well-dispersed micelle formation.

In the literature, the monomer of TEOS is hydrolyzed to be negatively charged, and the hydrolyzed TEOS interacts with positively charged CTAB micelles, leading to the formation of a mesoporous structure [7]. A faster hydrolysis rate has been reported in the fabrication of MSN in basic condition and, also, the type and amount of surfactant effect the hydrolysis and micellization of surfactant [40]. It has been reported that the high concentrations of silica precursors result in incomplete hydrolysis [20]. In this study, the use of small amounts of CTAB resulted in incomplete and small MSNs. This might be due to an increased ratio of alkoxide hydrolysis. In addition, herein, the MSN was not formed in acidic conditions, indicating the slow hydrolysis in acidic conditions. This is in line with the literature [7].

The surface area (Y_1_) of MSNs results from the formation of aligned pore structures due to the alkoxide hydrolysis of surfactants. The use of small amounts of CTAB increases the rate of alkoxide hydrolysis, resulting in incomplete and small MSNs. In accordance with the literature, the CTAB amount plays a key role in the surface area of MSN. It has been reported that the surface area of MSN shows a tendency to increase along with the increasing CTAB concentration (0.5–4% CTAB) [30,39,41]. In addition, herein, the surface area was increased by increasing the CTAB amount. High concentrations of silica precursors are incompletely hydrolyzed, resulting in reagent residues in the product and the production of silica walls, leading to low surface area [20]. As a result, it can be said that adjusting the ratio of CTAB and TEOS to an appropriate level is essential for manufacturing MSNs with large surface area. A large surface area can adsorb large amounts of drugs onto MSNs, increasing the amount of drug loaded.

Particle size (Y_2_) was found to increase the surface area by increasing the amount of silica precursor. The concentration of the silica precursor affects the rate of seed growth and nucleation. The amount of silica precursor acts as an important factor in the nuclear growth of MSNs, and the optimal range should be set to ensure high yield and desired particle size. It is possible to control the particle size of MSNs by adjusting the pH of the synthetic aqueous solution of MSNs. Changes in particle size by NaOH can lead to smaller particle formation due to condensation of TEOS, which has a slow hydrolysis rate at low pH. 

The zeta potential (Y_3_) can result in stable particle formation and distribution by forming well-dispersed micelles through a large amount of surfactant.

In conclusion, MSN composition was optimized through BBD, a response surface analysis method. In this study, only drug-free MSNs were optimized using the statistical approach (QbD), then the DOX was loaded into the optimized MSNs. In the case of MSNs, the drug is loaded into the mesopore, therefore, there is a limitation to assess the surface area after drug loading. Thus, the MSNs were optimized to maximize the surface area, minimize the particle size, and with the most negative zeta potential. Figure 2D shows the desirability obtained through optimization. Optimized formulations consisted of 0.617 g, 8.417 mL, and 2.726 mL of X_1_, X_2_, and X_3_, respectively. The desirability value of the optimized formulation was 0.697. Table 5 shows the predicted and observed values for optimized MSN. The optimized MSN formed uniform particles with a large surface area (Y_1_), particle size (Y_2_), and zeta potential (Y_3_) of 1165.2 m^2^/g, 116.1 nm, and −16.2 mV, respectively.

### 3.2. Physicochemical Properties of MSNs

#### 3.2.1. Morphology

**Figure 3:**
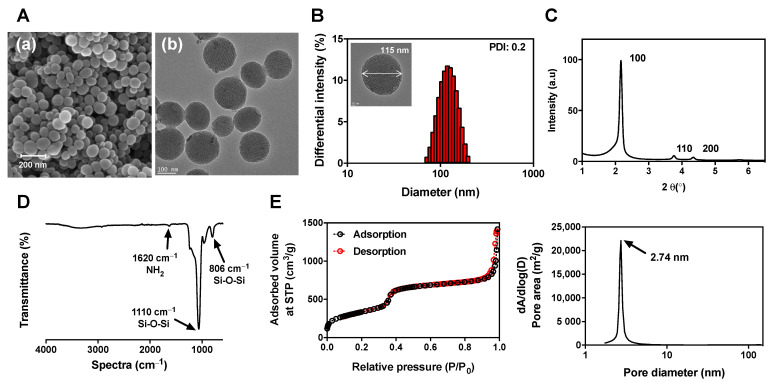
Physicochemical properties of MSNs. (**A**) (**a**) SEM image; (**A**) (**b**) TEM image; (**B**) particle size distribution; (**C**) SAXRD pattern; (**D**) FT-IR spectrum; (**E**) nitrogen adsorption–desorption isotherm curve, pore distributions.

The morphology of MSNs was observed in SEM and TEM images (Figure 3A). MSNs showed uniform particle distribution with a diameter of 110 to 120 nm. A well-ordered mesoporous structure with spherical particles has been reported as a characteristic of MCM-41-type MSNs [42]. According to DLS measurements, the mean hydrodynamic size of MSNs was 116.1 nm, and the PDI was 0.2 (Figure 3B). This is consistent with the size observed in SEM and TEM images.

#### 3.2.2. SAXRD

Figure 3C shows the MSN SAXRD pattern. It was confirmed that peaks appear at 2θ values of 100, 110, and 200 hkl in the SAXRD pattern of MSNs. It has been reported that the peak of the 2θ value represents the aligned hexagonal structure of MCM-41-type MSNs [42]. In addition, a well-ordered hexagonal pore structure of the MSNs was also observed in the TEM image.

#### 3.2.3. FT-IR Spectroscopy

In the FT-IR spectrum of MSNs, as shown in Figure 3D, strong absorption signals appeared at 806 and 1110 cm^−1^. These are peaks caused by asymmetric stretching and skeletal vibration of the Si–O–Si stretching, and it can be confirmed that silica ions form the skeleton for the Si–O–Si structure of MSNs [43]. 

#### 3.2.4. Nitrogen Adsorption–Desorption Isotherm

Figure 3E is the nitrogen adsorption–desorption isotherm and pore size distribution of MSNs. The pattern of the isotherm shown in Figure 3E indicates a type 4 isotherm that is free from the adsorption and desorption of gas through condensation of capillaries due to fine pores in the material having a mesostructure [44]. Therefore, it was confirmed that the optimized MSN is free of gas adsorption–desorption and has a well-developed mesostructure with a large surface area. Figure 3E was calculated using the standard BJH method, and it was confirmed that the uniform pore size was 2.74 nm.

#### 3.2.5. In Vitro Degradation

**Figure 4:**
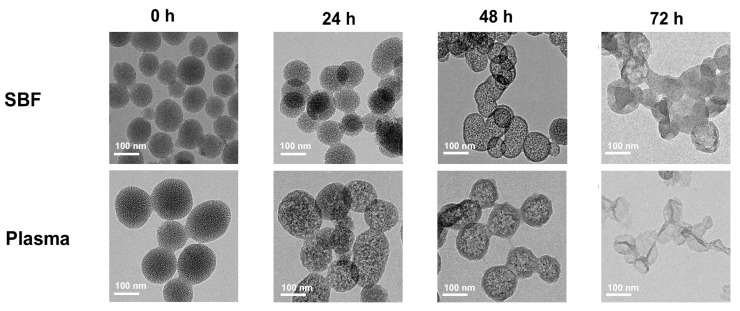
TEM images of MSNs in simulated body fluid (SBF) and rat plasma after 0, 24, 48, S and 72 h.

The degradation of MSNs was investigated through TEM. As can be seen in Figure 4, the degradation of MSNs was observed by collecting each sample at 0, 24, 48, and 72 h in both the SBF and rat plasma environments. As shown in Figure 4, MSNs at 0 h have an elaborate mesoporous structure. After 24 h immersion in SBF, the particle diameter of MSNs did not change, but some particles were connected to each other (Figure 4). After 48 h, the density of the aligned mesopores was weakened, and it was confirmed that the collapse of the structure proceeded (Figure 4). After 72 h, more particles agglomerated in MSNs, resulting in irregularly shaped structures, and no mesoporous structure specific to MSNs was observed (Figure 4). After immersion in plasma for 24 h, a collapse of the structure faster than the SBF environment was observed (Figure 4). After 48 h, the mesopore density of most MSNs had weakened (Figure 4). For 72 h, the MSN particles were mostly decomposed, and no particles were observed except for irregularly shaped substances (Figure 4). As the degradation of MSNs progresses, the silica density of the mesoporous structure of MSNs decreases and the surface becomes rough, indicating that the porous Si–O–Si framework of MSNs is attacked by water [45]. It is known that silica nanoparticles are dissolved by processes including hydration, hydrolysis, and ion exchange process [46]. Moreover, the plasma consists of various enzymes, such as the protease, if compared to the SBF. In addition, it has been reported that porous silicon nanoparticles (PSNs) show less stability in plasma than in PBS [47,48]. Silica, which is hydrolyzed in aqueous media, has been reported as nontoxic and to diffuse through the bloodstream to be cleared in urine [46]. As a result, the MSNs showed faster and stronger degradation in plasma than SBF, and the MSNs might be suitable for the purpose of sustained release. 

### 3.3. Drug Loading

**Figure 5:**
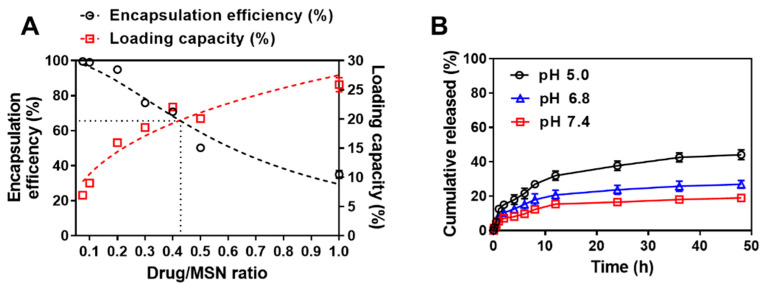
(**A**) Loading capacity and encapsulation efficiency according to DOX/MSN ratio; (**B**) the DOX release profile of MSN@DOX. Values are expressed as means ± SD (*n* = 3).

Using various concentrations of DOX in MSNs, the effect of DOX on the mass ratio was investigated through EE and LC. The DOX EE and LC of MSN@DOX at various mass ratios of MSNs and DOX are shown in Figure 5A. As the DOX/MSN mass ratio increased from 0.075 to 1, the encapsulation efficiency of the drug decreased from 99.52% to 34.94%. However, the drug loading capacity increased from 6.95% to 25.88%. Thus, in this study, 0.4 DOX/MSN mass ratio, which is the most efficient, was selected for the fabrication of MSNs.

### 3.4. In Vitro Release

The in vitro drug release pattern of MSN@DOX was confirmed at physiological cell pH (pH 7.4), weak tumor cell pH (pH 6.8), and tumor cell pH (pH 5.0). In Figure 5B, the rate of drug release from MSN@DOX was pH-dependent and increased with decreasing pH. The cumulative release of DOX could reach 52.77% and 38.97% after 48 h at pH 5.0 and pH 6.8, respectively, while the DOX was more slowly released at pH 7.4 (23.91%). In addition, DOX@MSN showed sustained release properties over 50 h. In this study, we aimed to develop and optimize the DOX@MSN for sustained release. However, regarding the formulation with sustained release, there is a chance of dose dumping in the clinical situation of daily intake. In the clinical situation, DOX is intravenously injected every 14–21 days. Thus, the patient treated with DOX@MSN would be unlikely to experience dose dumping compared to those treated with the daily intake formulation. The low drug release profile of DOX is due to the solubility of DOX decreasing as the pH increases. Decreases in pH increase the protonation of DOX due to its increased solubility, whereas the dissolution rate and solubility increase with increasing pH. It is also known that the slow release rate of the drug is due to the fact that the drug is contained in small pores and, therefore, does not easily diffuse into the medium [4]. Moreover, in the literature, it has been reported that the structure of MSNs (silica) is partially attacked under strongly acidic conditions [49]. Under acidic conditions, the loss of texture and structure has occurred due to the degradation of silicas. Although the degradation rate depends on the type of silica, the pores of MSNs are the first to be attacked by acid. Thus, in this study, the increase in DOX release under acidic conditions might be due to the acceleration of mesopore degradation.

DOX release from MSN@DOX is pH-dependent, and MSNs can act as pH-reactive nanocarriers. In a cancerous environment, acidic extracellular pH is one of the characteristics of tumor tissue. Thus, MSNs could be an effective drug carrier for cancer treatment.

### 3.5. Cytotoxicity Study

**Figure 6:**
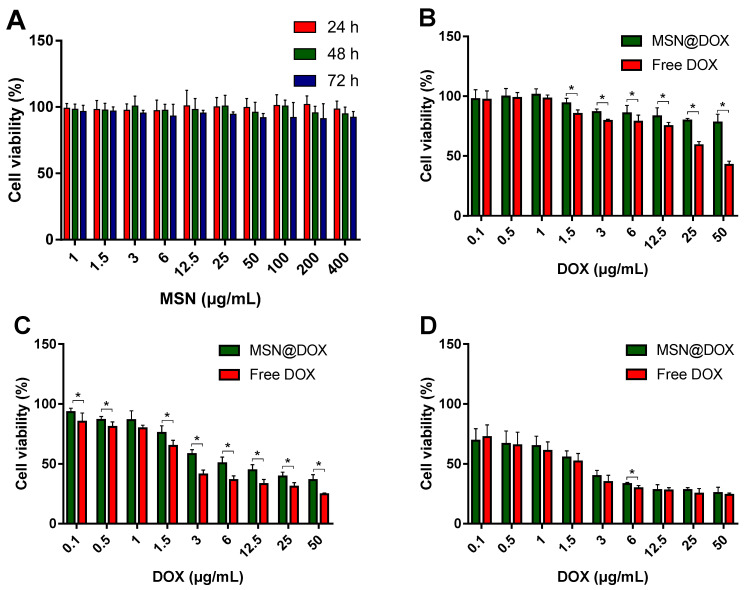
Cell viability of MCF-7 cells exposed to different concentration of MSNs, MSN@DOX, and free DOX. (**A**) MSNs; (**B**) after 24 h of MSN@DOX and free DOX; (**C**) 48 h of MSN@DOX and free DOX; (**D**) 72 h MSN@DOX and free DOX. Values are expressed as means ± SD (*n* = 5). * *p* < 0.05 compared with the free DOX group.

Cell viabilities of MSNs, MSN@DOX, and free DOX on MCF-7 cells were evaluated by MTT assay. The cytotoxicity test results of MSNs are shown in Figure 6A. MSNs did not show any cytotoxic effect on MCF-7 cells for 24, 48, and 72 h within the test concentration range. Even at high concentrations of 72 h, cytotoxicity was less than 10%, showing the negligible toxicity and excellent biocompatibility of MSNs. This aligned with previous research results that MSNs were less toxic and more biocompatible in MCF-7 cells [50]. On the other hand, an increase in the cytotoxicity of free DOX and MSN@DOX was observed with increasing time and drug concentration (Figure 6B–D). 

After 24 and 48 h, free DOX showed higher toxicity than MSN@DOX. However, when the incubation period was extended to 72 h, the cytotoxicity of MSN@DOX was significantly higher than that at 24 and 48 h, and the cytotoxicity was similar to that of free DOX at 72 h. This is because free DOX spreads rapidly into cells within a short period of time, due to the drug characteristics, and shows high cytotoxicity, whereas MSN@DOX continuously releases DOX molecules from MSNs [51]. The high cytotoxicity of early free DOX can lead to serious side effects due to the high concentration and long-term administration of chemotherapy drugs [52]. However, MSN@DOX can reduce the initial toxicity and deliver DOX to cancer cells in a continuous and controllable manner without side effects. These results suggest that MSN@DOX can be used as a safe and effective anticancer drug.

### 3.6. Cellular Uptake

**Figure 7:**
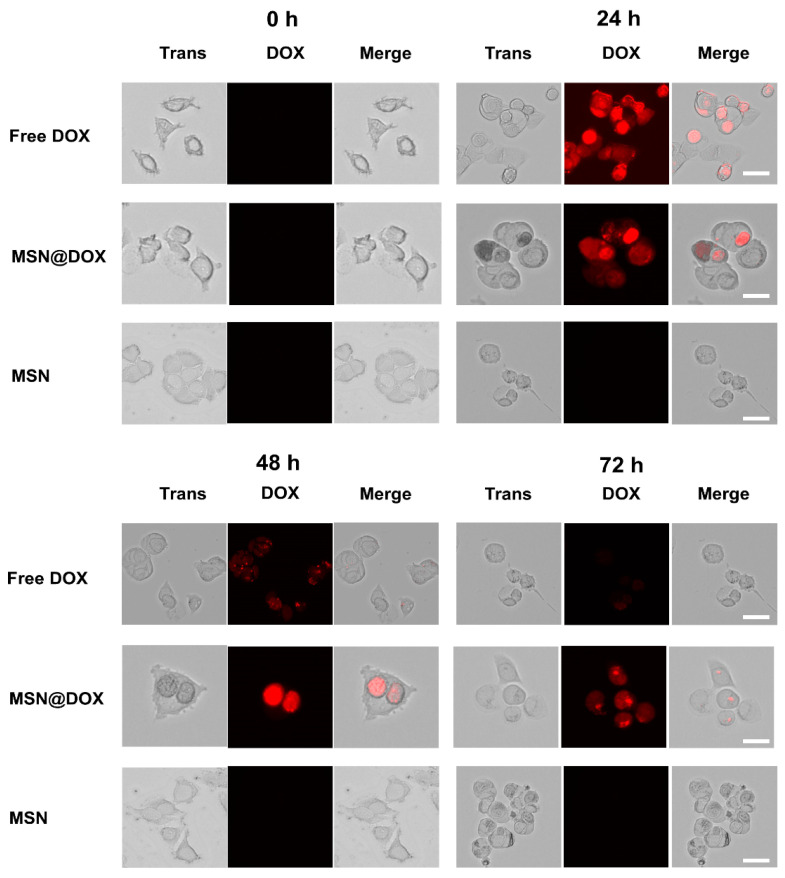
Representative fluorescence microscopy images of free DOX, MSN@DOX, and MSNs at 0, 24, 48, and 72 h in MCF-7 cells. Scale bar = 50 µm.

Cellular uptake of DOX from MSN@DOX, free DOX, and MSNs in MCF-7 cells was assessed by fluorescence microscopy. After the cells were incubated for 3 h, free DOX was rapidly absorbed into the cells by diffusion, as indicated by the strong fluorescence intensity in both the cytoplasm and nucleus. As shown in Figure 7, MSNs, MSN@DOX, and free DOX were internalized by MCF-7 cells. Cells incubated with MSNs did not show fluorescence at all time points. After incubation for 24 h, the fluorescence signal of free DOX was stronger than that of MSN@DOX, indicating rapid drug uptake. At 48 h, cells cultured with MSN@DOX showed obviously stronger DOX fluorescence in the nucleus until 72 h. On the other hand, the fluorescence signal of free DOX showed a maximum at 24 h and then decreased over 48 and 72 h. This is consistent with the toxicity test of MCF-7 cells, and MSN@ DOX showed cell uptake that sustained release of DOX from MCF-7 cells.

## 4. Conclusions

MSNs were successfully optimized through the statistical approach (BBD). Based on the results of SEM, TEM, SAXRD, FT-IR, nitrogen adsorption–desorption isotherm, and degradation tests, the optimized MSNs seem to be suitable for DOX delivery. In vitro release tests of MSN@DOX showed a sustained release profile, and the release rate increased as the pH value was decreased. Cytotoxicity studies showed that the MSNs had negligible cytotoxicity, while MSN@DOX showed significant cytotoxicity against MCF-7 cells. Cytotoxicity and fluorescence microscopy observations supported the potential of MSNs in sustained-release drug delivery systems. Thus, the optimized MSN@DOX could be an option to improve the bioavailability of DOX.

## Figures and Tables

**Table 1:** Factors and responses used in response surface design.

Response Surface Design		
Factors	Low Limit	High Limit
X_1_: CTAB amount (g)	0.25	1.00
X_2_: TEOS amount (mL)	5.00	10.00
X_3_: 2 N NaOH volume (mL)	1.75	7.00
Responses	Goal	
Y_1_: Surface area (m^2^/g)	Maximize	
Y_2_: Particle size (nm)	Minimize	
Y_3_: Zeta potential (mV)	Most negative	

**Table 2:** Preliminary experimental elements and summary.

Sample	CTAB (g)	TEOS (mL)	2 N NaOH (mL)	Particle Size (nm)	Structure Ordering
F1	0.125	5.00	3.50	-	△
F2	0.250	5.00	3.50	95.7 ± 1.0	○
F3	0.500	5.00	3.50	96.1 ± 4.4	○
F4	1.000	5.00	3.50	114.5 ± 8.6	○
F5	1.500	5.00	3.50	335 ± 128.8	○
F6	0.500	1.25	3.50	-	X
F7	0.500	2.50	3.50	-	X
F8	0.500	10.00	3.50	124.6 ± 13.5	○
F9	0.500	15.00	3.50	-	△
F10	0.500	5.00	0.88	-	X
F11	0.500	5.00	1.75	49.7 ± 2.7	○
F12	0.500	5.00	7.00	128.5 ± 10.1	○
F13	0.500	5.00	10.50	-	△

The notation of structural ordering is as follows: ○, good structural ordering; Δ, poor structural ordering; X, no structural ordering. In the case of F6, F7, and F10, samples were not generated and, thus, could not be measured for particle size analysis and SAXRD. Values are expressed as means ± SD (*n* = 3).

**Table 3:** Experiments configuration and observation response design. Values are expressed as means ± SD (*n* = 3).

Run	Factors			Response		
	X_1_	X_2_	X_3_	Y_1_	Y_2_	Y_3_
	CTAB (g)	TEOS (mL)	2 N NaOH (mL)	Surface Area (m^2^/g)	Particle Size (nm)	Zeta Potential (mV)
1	0.625	5.0	1.750	848.4 ± 217.6	75.5 ± 1.6	−15.5 ± 1.2
2	0.625	10.0	1.750	1091.0 ± 68.5	95.1 ± 6.9	−17.2 ± 3.6
3	0.250	10.0	4.375	956.4 ± 110.7	164.5 ± 2.8	−25.2 ± 3.2
4	1.000	7.5	7.000	1100.8 ± 110.9	223.2 ± 65.0	−9.3 ± 0.8
5	0.625	7.5	4.375	1204.3 ± 211.1	135.7 ± 6.5	−18.9 ± 2.7
6	0.625	7.5.	4.375	1202.2 ± 100.9	139.6 ± 10.9	−17.8 ± 0.5
7	0.250	7.5	7.000	919.1 ± 122.7	195.4 ±2.6	−25.7 ± 1.7
8	1.000	5.0	4.375	1112.7 ± 46.0	144.1 ± 13.2	−14.7 ± 1.9
9	0.625	7.5	4.375	1212.1 ± 97.4	140.5 ± 28.0	−19.4 ± 1.0
10	1.000	7.5	1.750	1029.4 ± 138.9	97.6 ± 14.3	−18.8 ± 0.1
11	0.250	7.5	1.750	818.5 ± 117.5	77.9 ± 1.2	−16.3 ± 4.4
12	0.625	7.5	4.375	1215.3 ± 89.0	140.7 ± 6.24	−18.2 ± 0.7
13	0.625	10.0	7.000	920.4 ± 267.1	243.4 ± 68.3	−20.1 ± 1.0
14	0.625	5.0	7.000	1107.9 ± 374.1	195.7 ± 5.1	−16.8 ± 3.4
15	0.250	5.0	4.375	828.6 ± 81.6	114.6 ± 2.3	−18.1 ± 5.8
16	1.000	10.0	4.375	1018.6 ± 128.7	175.4 ± 4.8	−13.7 ± 2.4
17	0.625	7.5	4.375	1229.4 ± 109.1	155.3 ± 1.8	−18.9 ± 2.8

**Table 4:** Summary of model fitting and statistical analysis.

Response	Suggested Model	Sequential *p*-Value	Lack of Fit *p*-Value	R^2^	Adjusted R^2^	Predicted R^2^	Adequate Precision
Y_1_	Quadratic	<0.0001	0.1340	0.9950	0.9886	0.9405	33.3058
Y_2_	Linear	<0.0001	0.4298	0.9762	0.9707	0.9600	41.1591
Y_3_	2FI	<0.0001	0.1503	0.9630	0.9408	0.8823	27.7873

**Table 5:** Predicted and actual values of the optimized MSNs. Values are expressed as means ± SD (n = 3).

Optimized Factors	Responses	95% CI Low Predicted Value	Predicted Value	95% CI High Predicted Value	Actual Value	Error Percentage (%)
X_1_: 0.617 g	Y_1_	1118.2	1158.4	1198.6	1165.2 ± 172.9	0.6
X_2_: 8.417 mL	Y_2_	91.3	110.3	129.3	116.1 ± 9.8	5.3
X_3_: 2.726 mL	Y_3_	−20	−17.8	−15.5	−16.2 ± 4.4	8.6

**Table 6:** Coefficient equations of responses according to the level of factors.

Responses	Coefficient Equations
Y_1_	1212.66 + 92.34X_1_ + 11.11X_2_ + 32.61X_3_ − 55.47X_1_X_2_ − 7.30 X_1_X_3_ − 107.53X_2_X_3_ − 129.29X_1_^2^ − 104.28 X_2_^2^ − 116.43X_3_^2^
Y_2_	147.89 + 11.00X_1_ + 18.56X_2_ + 63.94X_3_
Y_3_	−17.91+ 3.60X_1_ − 1.38X_2_ − 0.5062X_3_ + 2.03X_1_X_2_ + 4.75X_1_X_3_ − 0.3875X_2_X_3_

## Data Availability

Not applicable.
